# Influence of two different levels of intra-abdominal hypertension on bacterial translocation in a porcine model

**DOI:** 10.1186/2110-5820-2-S1-S17

**Published:** 2012-07-05

**Authors:** Torsten Kaussen, Pramod Kadaba Srinivasan, Mamdouh Afify, Christiane Herweg, René Tolba, Joachim Conze, Alexander Schachtrupp

**Affiliations:** 1 Department of Pediatric Cardiology and Intensive Care, University Children's Hospital, Hannover Medical School (MHH), Carl-Neuberg-Str. 1, 30625 Hannover, Germany; 2 Insitute of Laboratory Animal Science and Experimental Surgery, RWTH Aachen University, Pauwelsstr. 30, 52074 Aachen, Germany; 3 Department of Surgery, RWTH Aachen University, Pauwelsstr. 30, 52070 Aachen, Germany

**Keywords:** abdominal compartment syndrome, intra-abdominal hypertension, pneumoperitoneum, bacterial translocation, bowel, ischemia, histologic, mesenteric lymph node, pig, animal.

## Abstract

**Background:**

The purpose of the present study was to quantify bacterial translocation to mesenteric lymph nodes due to different levels of intra-abdominal hypertension (IAH; 15 vs. 30 mmHg) lasting for 24 h in a porcine model.

**Methods:**

We examined 18 anesthetized and intubated pigs (52.3 ± 4.7 kg) which were randomly allocated to three experimental groups (each *n *= 6) and studied over a period of 24 h. After preparation and establishing a steady state, the intra-abdominal pressure (IAP) was increased stepwise to 30 mmHg in six animals using a carbon dioxide (CO_2_) insufflator (IAP-30 group). In the second group, IAP was increased to 15 mmHg (IAP-15 group), while IAP remained unchanged in another six pigs (control group). Using a pulse contour cardiac output (PiCCO^®^) monitoring system, hemodynamic parameters as well as blood gases were recorded periodically. Moreover, peripheral and portal vein blood samples were taken for microbiological examinations. Lymph nodes from the ileocecal junction were sampled during an intra-vital laparotomy at the end of the observational period. After sacrificing the animals, bowel tissue samples and corresponding mesenteric lymph nodes (MLN) were extracted for histopathological and microbiological analyses.

**Results:**

Cardiac output decreased in all groups. In IAP-30 animals, volumetric preload indices significantly decreased, while those of IAP-15 pigs did not differ from those of controls. Under IAH, the mean arterial pressure (MAP) in the IAP-30 group declined, while MAP in the IAP-15 group was significantly elevated (controls unchanged). PO_2 _and PCO_2 _remained unchanged. The grade of ischemic damage of the intestines (histopathologically quantified using the Park score) increased significantly with different IAH levels. Accordingly, the amount of translocated bacteria in intestinal wall specimens as well as in MLN significantly increased with the level of IAH. Lymph node cultures confirmed the relation between bacterial translocation (BT) and IAP. The most often cultivated species were *Escherichia coli*, Staphylococcus, Clostridium, Pasteurella, and Streptococcus. Bacteremia was detected only occasionally in all three groups (not significantly different) showing gut-derived bacteria such as Proteus, Klebsiella, and *E. coli *spp.

**Conclusion:**

In this porcine model, a higher level of ischemic damage and more BT were observed in animals subjected to an IAP of 30 mmHg when compared to animals subjected to an IAP of 15 mmHg or controls.

## Introduction

Abdominal compartment syndrome (ACS) is characterized by intra-abdominal hypertension (IAH) accompanied by new failure of at least one organ system [1]. Mortality has been reported to be as high as 50% even after operative decompression [2] due to multi-organ failure (MOF) [2]. In most cases, the first clinical signs of organ impairment appear about 24 h after the onset of IAH or its underlying cause, respectively [2]. At this point, IAH often progresses fluently to ACS. Several investigative groups revealed an IAH-induced restriction of cardiac output and organ perfusion, as well as venous outflow obstruction, culminating in congestion and ischemia [3-6]. Stasis and edema themselves lead to an additional increase in IAP peaking in a vicious circle. Using a pig model, our study group described consecutive histomorphological lesions of nearly all intrathoracic and intra-abdominal organs as early as 6 h after the onset of IAH [7]. Focusing on the gastrointestinal tract, IAH experimentally results in increasing mucosal and bowel wall damage [8,9]. It is well known that mesenteric ischemia per se, regardless of the IAP, may lead to decreased integrity, increased permeability, bacterial translocation (BT), and the development of MOF [10-12].

Rezende-Neto et al. as well as Kubiak et al. experimentally found increased pro-inflammatory cytokines secondary to increased intra-abdominal pressure (IAP) [13,14]. To what extent BT is relevant for this systemic inflammation and in total for the pathogenesis of MOF if induced by IAH has not yet been clarified in all regards. Indeed, several study groups depicted the appearance of BT in small-animal IAH models [15-20]. So far, in large-animal models, it has not been shown that IAH will lead to BT. This could be due to the fact that the duration of IAH was not long enough.

Thus, the present study aimed to investigate whether BT might be detected in an established large-animal model of IAH lasting for 24 h. To distinguish between time- and pressure-dependent causes and consequences, pigs were exposed to two different levels of IAH (15 and 30 mmHg, respectively).

## Materials and methods

All experiments were performed in accordance with the German legislation governing animal studies following the *Principles of Laboratory Animal Care *(National Institutes of Health publication 85-23, revised 1996 [21]). Official permission was granted from the governmental animal care office (Landesamt für Natur, Umwelt und Verbraucherschutz Nordrhein-Westfalen, Recklinghausen, Germany). Eighteen male German landrace pigs from a disease-free barrier breeding facility were housed in ventilated rooms and allowed to acclimatize to their surroundings for a minimum of 5 days before surgery. The animals, weighing 52.3 ± 4.7 kg (mean ± standard deviation (SD)) (range, 47 to 58 kg), were fasted 24 h prior to the experiments with free access to water and randomly allocated to one of the three experimental groups (each consisting of *n *= 6 animals). Due to the fact that up to 90% of clinically healthy pigs suffer from occult bronchitis and pneumonia [22,23], all animals received a single shot of 2 g of amoxicillin i.m. as antibiotic prophylaxis 48 h before surgery.

Initially, each animal was intramuscularly administered 250 mg fluoperidol (Stresnil^® ^10%, Janssen Pharmaceutica, Beerse, Belgium) and 10 mg atropine (premedication). About 10 min later, 500 mg ketamine and 3.5 mg/kg body weight pentobarbital (Narcoren^®^, Merial GmbH, Hallbergmoos, Germany) were given prior to intubation. Animals were ventilated volume cycled with a Servo 900C Respirator (Siemens, Solna, Sweden) (FiO_2_, 25%; PEEP, 5 cm H_2_O). General anesthesia was maintained with an average of 8 mg·h^-1 ^ketamine and 2.5 mg·h^-1 ^pentobarbital. Under these medications, no animal showed vegetative alterations as indirect signs of insufficient analgesia or sedation. At baseline, the respiratory rate was set to 15 breaths/min. The tidal volume was adjusted to maintain a PCO_2 _of 35 to 40 mmHg in each animal. These parameters were kept unchanged during the whole experimental period. Blood gas analysis was carried out every 4 h. Normal saline was infused at a constant rate of 1.5 mL·kg^-1^·h^-1 ^throughout the examination. Animals were in supine position during the whole investigation.

A 4-French thermistor-tipped catheter (PV 2016L20, PULSION Medical Systems, Munich, Germany) for transpulmonary single-indicator dilution measurement was placed in the descending aorta via the femoral artery and connected to a pulse contour cardiac output (PiCCO^®^) monitoring unit (PULSION Medical Systems). Further catheters were placed into the carotid artery and superior vena cava. A core temperature of 36°C to 38°C was maintained by the application of a thermal mattress. Urine output was recorded via suprapubic catheterization. Finally, a laparoscopy was performed to exclude intra-abdominal hemorrhage. After release of the pneumo-peritoneum, animals were allowed to stabilize.

After 1 h of steady-state phase, measurement of baseline values was performed. Afterwards, CO_2 _was insufflated to increase the IAP to 15 mmHg in six animals (IAP-15 group) and to 30 mmHg in another six animals (IAP-30 group). IAP was increased in steps of 5 mmHg with regard to the insufflator readings until the target pressure of 15 or 30 mmHg, respectively, was achieved and was maintained throughout the examination using this automatically controlled insufflator (Electronik-pneu, Karl Storz, Tuttlingen, Germany). Six animals with an unchanged IAP served as controls. After zeroing at the level of the mid-axillary line with the pig being positioned supine, measurements of the intravesical pressure (IVP) were performed hourly by injecting 50 mL of saline into the bladder after the catheter system had been flushed. The resulting end-expiratory pressure was measured using a pressure transducer and a monitoring system (Sirecust 404, Siemens, Munich, Germany).

To avoid postmortem changes, an intra-vital laparotomy was performed at the end of the observational period of 24 h. One lymph node from the ileocecal junction was taken under sterile conditions and immediately fixed for histological analysis. Furthermore, a blood sample from the portal vein was gained under sterile conditions. Thereafter, animals were killed by an overdose of pentobarbital, and histological specimens were taken from the small and large bowel (150 cm cranially and 120 cm distally from the ileocecal junction) including the corresponding mesenteric lymph nodes (MLN).

### Hemodynamic, respiratory, and functional parameters

Using transpulmonary thermodilution measurement (PiCCO^®^, PULSION Medical Systems, Munich, Germany), the following hemodynamic parameters and 'filling volumes' were investigated: cardiac index (CI = cardiac output related to the body surface area), mean arterial pressure (MAP), global end-diastolic volume index (GEDVI), and extravascular lung water index (EVLWI). CI and MAP were continuously surveyed via pulse contour analysis, while the other above-mentioned parameters were measured hourly [24,25]. Furthermore, heart rate (HR), central venous pressure (CVP), and urine output (UO) were recorded (AS/3, Datex Ohmeda, Helsinki, Finland). Afterwards, abdominal perfusion pressure (APP = MAP - IAP) and renal filtration gradient (RFG = MAP - 2·IAP) were calculated [1]. Every 2 h, blood gas analyses were performed.

### Light microscopy

Histological specimens of bowel and MLN were treated and stained following standard staining protocols (hematoxylin and eosin, periodic acid Schiff, Gram's stain). Afterwards, the specimens were examined light microscopically (each 10 high-power fields (HPF)) for ischemia (bowel) and for the presence of bacteria (bowel and MLN) by a single pathologist (MA) blinded for the identity of the specimens with the help of a LEICA DM 2500 (LEICA, Wetzlar, Germany). The mucosal damage of the bowel was graded using the Park score (Table 1[26]). Regarding lymph nodes, the number of bacteria was assessed by counting single bacteria (high-power magnification: maximum ×400) or groups of bacteria when further differentiation was not possible using oil immersion lens (maximum magnification, ×1,000). The extent of BT through the intestinal wall was analyzed semi-quantitatively using a histopathological classification score (Table 2).

**Table 1:** Histopathological classification of ischemic damage of the intestinal wall according to Park et al. [26]

Park score	Histopathological appearance/damage of bowel wall
Grade 0	Normal mucosa
Grade 1	Subepithelial space at villous tip
Grade 2	More extended subepithelial space
Grade 3	Epithelial lifting along villus sides
Grade 4	Denuded villi
Grade 5	Loss of villous tissue
Grade 6	Crypt layer infarction
Grade 7	Transmucosal infarction
Grade 8	Transmural infarction

**Table 2:** Histopathological classification of bacterial translocation through the intestinal wall

Bowel wall translocation score	Histopathological appearance (bacterial count in 10 HPF)
Grade 0	No bacteria detectable (neither in mucosa nor in submucosa)
Grade 1	Few bacteria in the mucosa (up to five findings in 10 HPF)
Grade 2	Many bacteria in the mucosa (more than five findings in 10 HPF)
Grade 3	Bacteria in mucosa as well as in submucosa

### Microbiological analysis

Under sterile conditions, lymph nodes were removed from the ileocecal junction, immediately frozen using liquid nitrogen, and stored at -70°C until further analysis. After thawing the lymph nodes, calculation of wet weight was performed, and specimens were dissected and submerged in thioglycolate broth (Oxoid, Wesel, Germany). After homogenization, dilution series were made and aliquots plated on Columbia blood, McConkey, Schaedler, and Kanamycin-Vancomycin agars (BD Corp., Heidelberg, Germany). After an incubation period of up to 72 h (37°C), resulting colony forming units (CFU) were counted, subcultivated, and identified according to standard microbiological procedures (API20E-systems, Fa. API Biomerieux SA, Marcy l'Etoile, France). Results are given as CFU per gram wet tissue.

Additional to portal vein blood sampling, peripheral venous blood samples were drawn at baseline as well as 8, 16, and 24 h after the onset of IAH. All blood samples were taken under sterile conditions and inoculated for 7 days using customary blood culture bottles (BacTec^®^Plus+ and BacTec^®^Lytic/10, Fa. Becton-Dickinson, BENEX, Shannon, Ireland) and the BacTAlert^®^-detection System (BACTEC9000, Organon Teknika, Eppenheim, Germany [27]). When bacterial growth was detected, aliquots were plated on Columbia blood, McConkey, Schaedler, and Kanamycin-Vancomycin agars (BD, Heidelberg, Germany) and further differentiated.

### Statistical analysis

Data were analyzed for normal distribution according to the Shapiro-Wilk test. In the presence of normal distribution, results are presented as mean ± SD. Further statistical analysis was carried out using an analysis of variance for repeated measurements (ANOVA) combined with a post hoc test according to the Tukey method. Within each group, a paired *t *test was performed between values of the baseline and the following measurements beginning after 24 h. Furthermore, unpaired *t *tests were performed between the experimental groups and the controls at corresponding moments, again beginning with the values after 24 h. A *P *< 0.05 was considered significant. In the case of repeated (*n*) pairwise *t *testing, the level of significance was adjusted to *P *< 0.05·*n*^-1 ^according to the Bonferroni correction.

If appropriate, the Pearson correlation coefficient 'R' was calculated in order to check the strength of linear dependence between two parameters. R ranges from -1 to 1. A value of 0 implies that there is no linear correlation, while a value of +1 or -1 implies that a linear equation describes the relationship.

**Figure 1:**
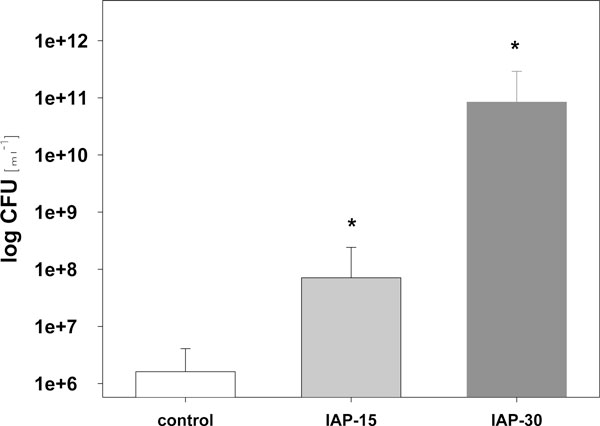
**CFU in ileocecal MLN of pigs exposed to IAH for 24 h**. Mean ± standard error of the mean of cultivable CFU in mesenteric lymph nodes taken from the ileocecal region according to the different levels of IAP (IAP-15/IAP-30: animals with an IAP of 15 mmHg or 30 mmHg, respectively, lasting for 24 h). Please take into consideration the logarithmic scale of the axis of ordinates. Asterisk denotes significant difference to control (IAP-15: *p *< 0.04; IAP-30: *p *< 0.01).

The graded results of histological data are presented as median (minimum to maximum). To detect differences between the experimental groups and the control, the Mann-Whitney *U *test was applied. To simplify the illustration of bar graphs of cultivable CFU, values in Figure 1 are depicted as means with standard errors of the mean. Statistical analysis was calculated using Statistical Package for Social Sciences (SPSS) 12.0.1 for Windows (SPSS Inc., Chicago, Illinois, USA).

## Results

### Hemodynamic, respiratory, and functional parameters

HR did not change within the groups nor did a difference occur between the study groups and controls (range, 84.3 (SD 36.3) to 138.3 (SD 15.7) min^-1^). Further results of the hemodynamic and respiratory monitoring are depicted in Tables 3, 4, and 5. Pearson's correlation coefficient revealed a strong correlation between base excess (BE) and GEDVI (Pearson's R after 24 h, 0.73).

**Table 3:** Hemodynamic parameters at intervals of 6 h

Parameter	Group	0 h	6 h	12 h	18 h	24 h	Unit
CI^a^	IAP-30^bc^,	110.6 ± 21.3	62.0 ± 25.5^bc^,	44.8 ± 19.2^bc^,	39.9 ± 18.7^bc^,	22.5 ± 12.7^bc^,	mL·min^-1^·m^-2^
	IAP-15^bc^,	122.7 ± 43.9	95.9 ± 20.3	83.8 ± 15.1	74.9 ± 13.4^b^	71.1 ± 17.9^b^	L·min^-1^·m^-2^
	Control^bc^,	110.7 ± 19.7	94.6 ± 11.4	75.9 ± 9.2^b^	73.7 ± 7.3^b^	82.8 ± 17.1^b^	L·min^-1^·m^-2^
CVP^a^	IAP-30^bc^,	5.2 ± 2.0^bc^,	13.0 ± 2.4^bc^,	11.5 ± 2.0^bc^,	12.0 ± 2.7^bc^,	12.5 ± 2.7^bc^,	mmHg^bc^,
	IAP-15^bc^,	4.7 ± 1.9^bc^,	9.5 ± 2.0^bc^,	9.9 ± 1.8^bc^,	10.6 ± 4.4^bc^,	9.7 ± 2.7^bc^,	mmHg^bc^,
	Control^bc^,	4.1 ± 3.0^bc^,	2.6 ± 2.0^bc^,	1.9 ± 1.6^bc^,	2.8 ± 2.2^bc^,	3.2 ± 1.6^bc^,	mmHg^bc^,
EVLWI^a^	IAP-30^bc^,	2.7 ± 1.3	3.6 ± 1.6	4.1 ± 2.1	4.9 ± 2.0	7.3 ± 2.4^bc^,	mL·kg^-1^
	IAP-15^bc^,	2.5 ± 1.1	4.4 ± 1.8	5.0 ± 1.2	5.2 ± 2.1	5.2 ± 1.3^bc^,	mL·kg^-1^
	Control	2.2 ± 0.8	2.6 ± 0.5	3.4 ± 0.8	3.2 ± 0.7	3.1 ± 1.2	mL·kg^-1^
GEDVI	IAP-30^bc^,	8.2 ± 1.3	6.1 ± 1.4	5.5 ± 1.7^b^	5.8 ± 1.5^bc^,	4.0 ± 1.7^bc^,	mL·m^-2^
	IAP-15^bc^,	9.5 ± 2.6	9.8 ± 2.0	9.6 ± 2.2	8.0 ± 0.7	9.0 ± 1.9	mL·m^-2^
	Control	8.4 ± 1.4	8.4 ± 1.4	9.4 ± 1.2	9.4 ± 2.4	10.1 ± 1.1^b^	mL·m^-2^

^a^Significant change during 24 h according to ANOVA and post-hoc analysis: CI (*P *= 0.004); CVP (*P *< 0.001); EVLWI (*P *= 0.033). ^b^Significant difference from baseline according to paired *t *test: CI (IAP-30, 6 h: *P *= 0.002; IAP-15, 18 h: *P *= 0.024; control, 12 h: *P *= 0.004); CVP (IAP-30, 6 h: *P *< 0.001 and IAP-15, 12 h: *P *= 0.002); EVLWI (IAP-30, 24 h: *P *= 0.017 and IAP-15, 24 h: *P *= 0.013); GEDVI (IAP-30, 18 h: *P *= 0.026 and control, 24 h: *P *= 0.038). ^c^Significant difference from corresponding control according to unpaired *t *test: CI (IAP-30, 6 h: *P *= 0.002); CVP (IAP-30, 6 h: *P *= 0.002 and IAP-15, 12 h: *P *= 0.002); EVLWI (IAP-30; 24 h: *P *= 0.014 and IAP-15, 24 h: *P *= 0.019); GEDVI (IAP-30, 18 h: *P *= 0.002). IAP-15/IAP-30: animals exposed to an IAP of 15 mmHg or 30 mmHg, respectively, lasting for 24 h (each group consisting of six animals).

**Table 4:** Results of MAP and associated parameters at intervals of 6 h

Parameter	Group	0 h	6 h	12 h	18 h	24 h	Unit
MAP^ab, c^,	IAP-30^bc^,	72.6 ± 8.1^bc^,	70.8 ± 18.7^bc^,	60.8 ± 17.8^bc^,	52.9 ± 12.1^c^	45.0 ± 14.6^bc^,	mmHg^bc^,
	IAP-15^bc^,	75.2 ± 14.5^bc^,	77.1 ± 9.8^bc^,	88.7 ± 10.2^bc^,	91.8 ± 18.7^bc^,	90.5 ± 17.3^b^	mmHg^bc^,
	Control^bc^,	71.5 ± 12.9^bc^,	70.1 ± 7.7^bc^,	80.4 ± 14.1^bc^,	82.7 ± 8.0^bc^,	83.6 ± 10.2^bc^,	mmHg^bc^,
APP^a^ = MAP - IAP	IAP-30^bc^,	67.4 ± 8.3	58.3 ± 11.0	49.3 ± 18.3^bc^,	43.3 ± 17.1^bc^,	32.5 ± 16.5^bc^,	mmHg^bc^,
	IAP-15^bc^,	70.5 ± 13.8	69.4 ± 8.0	78.8 ± 10.1	79.7 ± 12.1	80.8 ± 16.8	mmHg^bc^,
	Control^bc^,	67.0 ± 14.0	67.0 ± 7.1	78.5 ± 13.8	80.1 ± 10.5	80.4 ± 10.2	mmHg^bc^,
RFG^a^ = MAP - 2·IAP	IAP-30^bc^,	72.6 ± 8.1	13.0 ± 9.6^bc^,	7.8 ± 7.2^bc^,	4.6 ± 6.6^bc^,	0.0 ± 0.0^bc^,	mmHg^bc^,
	IAP-15^bc^,	75.2 ± 14.5	47.7 ± 8.1^bc^,	58.7 ± 10.2^bc^,	62.4 ± 16.4^bc^,	60.5 ± 17.3^bc^,	mmHg^bc^,
	Control	71.5 ± 12.9	70.1 ± 8.0	80.4 ± 14.1	82.8 ± 9.9	83.6 ± 10.2	mmHg^bc^,

^a^Significant change during 24 h according to ANOVA and post-hoc analysis: MAP (*P *= 0.037); APP (*P *= 0.002); RFG (*P *= 0.001). ^b^Significant difference from baseline according to paired *t *test: MAP (IAP-30, 24 h: *P *= 0.014 and IAP-15, 24 h: *P *= 0.010); APP (IAP-30, 12 h: *P *= 0.026); RFG (IAP-30, 6 h: *P *= 0.007 and IAP-15, 6 h: *P *= 0.046). ^c^Significant difference from corresponding control according to unpaired *t *test: MAP (IAP-30, 18 h: *P *= 0.004); APP (IAP-30, 12 h: *P *= 0.001); RFG (IAP-30, 6 h: *P *= 0.003 and IAP-15, 6 h: *P *= 0.011). IAP-15/IAP-30: animals exposed to an IAP of 15 mmHg or 30 mmHg, respectively, lasting for 24 h (each group consisting of six animals).

**Table 5:** Results of blood gas analyses at intervals of 6 h

Parameter	Group	0 h	6 h	12 h	18 h	24 h	Unit
pH^ab^,	IAP-30^ab^,	7.40 ± 0.07	7.38 ± 0.04	7.31 ± 0.09	7.28 ± 0.10	7.22 ± 0.13^ab^,	
	IAP-15^ab^,	7.43 ± 0.05	7.36 ± 0.10	7.47 ± 0.04	7.47 ± 0.05	7.46 ± 0.04	
	Control^ab^,	7.45 ± 0.09	7.45 ± 0.07	7.51 ± 0.04	7.48 ± 0.07	7.48 ± 0.05	
BE^c^	IAP-30^ab^,	1.1 ± 4.3	-0.8 ± 2.5	-5.6 ± 6.8	-6.8 ± 5.9^ab^,	-10.4 ± 4.6^ab^,	mmol·L^-1ab^,
	IAP-15^ab^,	-0.5 ± 4.1	-2.1 ± 3.8	3.0 ± 1.9	2.2 ± 2.9	1.1 ± 2.7	mmol·L^-1ab^,
	Control^ab^,	1.8 ± 2.3	3.2 ± 3.0	4.9 ± 1.4	3.6 ± 2.0	3.7 ± 1.2	mmol·L^-1ab^,
PO_2 _	IAP-30^ab^,	137.3 ± 30.8	124.7 ± 11.1	121.27 ± 23.5	116.0 ± 18.3	105.2 ± 31.0	mmHg^ab^,
	IAP-15^ab^,	144.9 ± 17.1	141.2 ± 18.7	132.8 ± 9.7	135.6 ± 37.1	117.3 ± 40.1	mmHg^ab^,
	Control^ab^,	131.5 ± 20.8	142.9 ± 23.1	128.5 ± 18.2	146.6 ± 32.7	130.4 ± 14.2	mmHg^ab^,
PCO_2_	IAP-30^ab^,	41.6 ± 3.6	41.9 ± 4.5	37.2 ± 10.2	33.2 ± 11.9	31.1 ± 13.4	mmHg^ab^,
	IAP-15^ab^,	35.5 ± 3.8	38.8 ± 5.9	37.6 ± 2.9	35.4 ± 4.9	34.6 ± 4.9	mmHg^ab^,
	Control	38.4 ± 5.8	36.9 ± 7.0	36.7 ± 4.4	36.3 ± 7.5	37.1 ± 5.2	mmHg^ab^,

^a^Significant difference from baseline according to paired *t *test: pH (IAP-30, 24 h: *P *= 0.017); BE (IAP-30, 18 h: *P *= 0.025). ^b^Significant difference from corresponding control according to unpaired *t *test: pH (IAP-30, 18 h: *P *= 0.005); BE (IAP-30, 18 h: *P *= 0.036). ^c^Significant change during 24 h according to ANOVA and post-hoc analysis: BE (*P *= 0.019). IAP-15/IAP-30: animals exposed to an IAP of 15 mmHg or 30 mmHg, respectively, lasting for 24 h (each group consisting of six animals).

Under the condition of elevated IAP, UO significantly decreased from 1.32 mL·h^-1^·kg^-1 ^(baseline) to 0.27 mL·h^-1^·kg^-1 ^after 4 h in the IAP-30 group (*P *< 0.001). After 24 h, all animals were anuric. UO of the IAP-15 group significantly dropped from 1.66 mL·h^-1^·kg^-1 ^(baseline) to 1.27 mL·h^-1^·kg^-1 ^after 24 h (*P *= 0.008). In contrast, UO of the controls significantly increased from 1.42 mL·h^-1^·kg^-1 ^(baseline) to 3.03 mL·h^-1^·kg^-1 ^after 24 h (*P *= 0.0015).

As soon as IAP was increased, a strong correlation between UO and RFG could be calculated (range of Pearson's R, 0.72 to 0.90).

### Light microscopy

**Figure 2:**
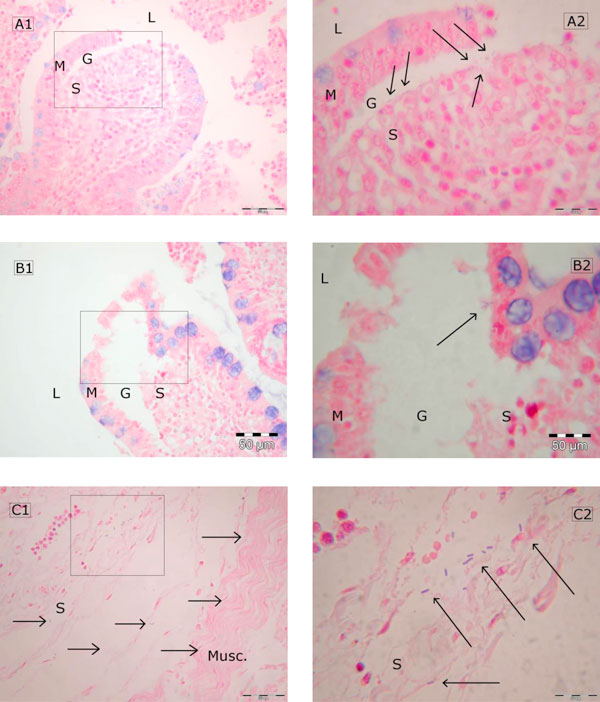
**Translocated bacteria in bowel wall specimens of pigs exposed to IAH for 24 h**. Histological specimens (Gram-stained) of bowel walls of pigs exposed to an IAP of 15 mmHg (***B***) and 30 mmHg (***A***, ***C***) for 24 h. Boxes in A1, B1, and C1 (magnification: each ×400) mark extracts which are magnified in A2, B2, and C2 (magnification: each ×1,000). Bowel wall layers are labeled 'M' (tunica mucosa), 'S' (tela submucosa), and 'Musc' (tunica muscularis). 'L': intestinal lumen, 'G': Gruenhagen's space (according to Park's classification [26], ischemic damage to the gut is accompanied by proportionally increasing lifting of epithelia. In this way, the developing space between the mucosa and submucosa is named after Gruenhagen). Histomorphological damage shown in A and B correlates to Park score 1 (B (IAP-15): subepithelial space at villous tips) and 2 (A (IAP-30): extended subepithelial spaces). Cocci as well as rods passed Gruenhagen's space (see arrows) and invaded the tela submucosa (A, B: translocation score 2 to 3). Bacteria shown in C even crossed the tela submucosa and started invading the tunica muscularis (see arrows).

In the IAP-30 group, the small and large bowel mucosa presented signs of moderate ischemic damage (Figure 2, A1 and B1). In the presence of an IAP of 15 mmHg, the bowel mucosa was less severely, but still significantly, damaged when compared to that of the controls (Table 6).

**Table 6:** Ischemic damage of the small and large bowel in pigs exposed to IAH for 24 h

Localization	Control (C)	IAP-15	IAP-30	*P*-value (C vs. IAP-15)	*P*-value (C vs. IAP-30)
Small bowel (Park score)	1 (1 to 2)	2 (1 to 4)^a^	4 (2 to 6)^a^	0.0001	0.0001
Large bowel (Park score)	1 (1 to 2)	1 (1 to 2)^a^	3 (2 to 5)^a^	0.0001	0.0001

Ischemic bowel in pigs exposed to an IAH for 24 h according to the Park score (Table 1, [26]). ^a^Significantly different from control according to Mann-Whitney *U *test. IAP-15/IAP-30: animals exposed to an IAP of 15 mmHg or 30 mmHg, respectively (each group consisting of six animals).

#### Bacterial count in the intestinal wall

In the presence of IAH, mucosal and submucosal specimens of the bowel displayed more translocated bacteria (Figure 2). BT was also found in the specimen of pigs belonging to the control group (grade 1 (0 to 2)). The IAP-30 group showed significantly more BT (*P *= 0.04; Table 7; grade 2 (1 to 3)). In IAP-15, the median score was 1 (1 to 2) (not significantly different (n.s.) compared to controls). The highest counts of bacteria were found in specimens of the intestinal wall around the ileocecal valve, regardless of the treatment group.

**Table 7:** Bacterial counts in bowel wall specimen of pigs exposed to IAH for 24 h

Localization	Control (C)	IAP-15	IAP-30	*P*-value (C vs. IAP-15)	*P*-value (C vs. IAP-30)
Ileum	1 (1 to 2)	1 (1 to 1)	1.5 (1 to 3)	n.s.	n.s.
Ileocecal junction	1.5 (1 to 2)	1 (1 to 2)	2 (1 to 3)	n.s.	n.s.
Cecum	1 (0 to 1)	1 (1 to 1)	1 (1 to 2)	n.s.	n.s.
Overall	1 (0 to 2)	1 (1 to 2)	2 (1 to 3)^a^	n.s.	0.044

Translocated bacteria in the ileum, ileocecal junction, and cecum of pigs exposed to IAH for 24 h according to translocation score (referring to 10 high-power fields): grade 0: no bacteria in the mucosa and submucosa, grade 1: bacteria in up to 5 of 10 high-power fields of the mucosa, grade 2: bacteria in more than 5 of 10 high-power fields of the mucosa, and grade 3: bacteria in the mucosa and even submucosa. ^a^Significant difference to control according to Mann-Whitney *U *testing. IAP-15/IAP-30: animals exposed to an IAP of 15 mmHg or 30 mmHg, respectively, lasting for 24 h (each group consisting of six animals).

#### Bacterial counts in mesenteric lymph nodes

**Figure 3:**
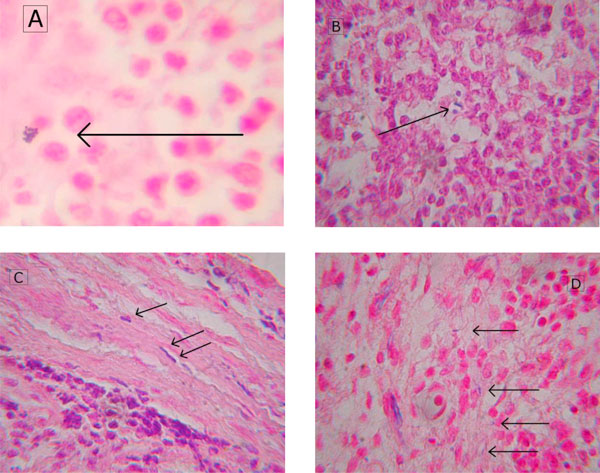
**Translocated bacteria in MLN specimens of pigs exposed to IAH for 24 h**. Histological specimens (Gram-stained, each ×1,000) of MLNs of pigs exposed to an IAP of 15 mmHg (**D**) and 30 mmHg (**A**, **B**, **C**) for 24 h. Cocci (A, D) as well as rods (B, C, D) were detectable (see arrows).

Using Gram's stain, cocci as well as rods were detectable in MLN of all three groups. In controls as well as in both experimental groups, Gram-positive bacteria predominated slightly when compared to the amount of Gram-negative germs (Figure 3).

**Figure 4:**
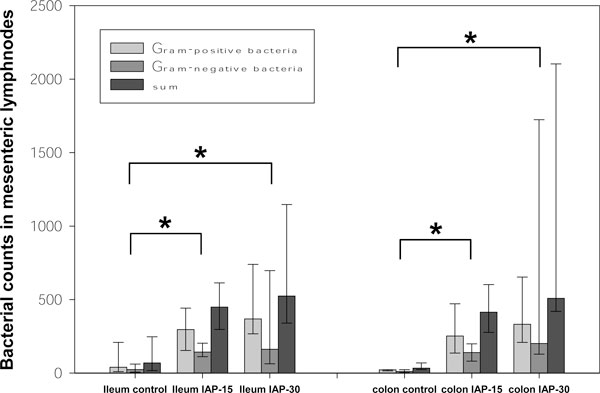
**Bacterial counts in MLN of pigs exposed to IAH for 24 h**. Median (minimum to maximum) of bacterial counts per mesenteric lymph node of the small and large bowel (ileum and colon) of pigs exposed to an IAP of 15 mmHg (IAP-15) or 30 mmHg (IAP-30) for 24 h. Results given as sum as well as distinguished between Gram-positive and Gram-negative bacteria. Asterisk denotes significant difference between bacterial counts in MLN of treatment group pigs and controls (*P *< 0.05).

Bacterial count in MLN was significantly higher in both test groups when compared to those of control specimens (Figure 4). Against controls, the count of Gram-positive bacteria was about nine times higher in the IAP-30 group and seven times higher in IAP-15 group. Concerning Gram-negative bacteria, the count was about seven times higher in the IAP-30 group and six times higher in the IAP-15 group (Figure 1).

### Microbiological analysis of ileocecal mesenteric lymph nodes

In the IAP-30 group, the number of CFU was more than 50,000 times higher than in controls. CFU of IAP-15 animals outnumbered those of controls 44-fold.

Aerobic as well as anaerobic bacteria were cultivable (Table 8). There was no difference between the groups regarding the kind of bacteria.

**Table 8:** Microbiologically detected bacterial species in pigs exposed to IAH for 24 h

Kinds of microbiologically detected bacteria	
Mesenteric lymph nodes (MLN)	Portal vein blood (PVB)
*E. coli *spp. Staphylococcus spp. Clostridium spp. Pasteurella spp. Streptococcus spp.	*E. coli *spp. *P. mirabilis* *K. pneumoniae*

### Blood cultures

Blood cultures gained from peripheral venous blood samples stayed sterile in all groups. Portal venous blood (PVB) cultures were occasionally positive in all three groups without displaying any difference. In contrast to MLN cultures, Gram-positive bacteria could not be detected (Table 8).

## Discussion

In humans, ACS is defined as IAH (sustained IAP > 20 mmHg in adults [1] and IAP > 10 mmHg in children [28], respectively) in combination with new ongoing organ failure. Useful tools to quantify organ dysfunctions are statistical models such as the Sequential Organ Failure Assessment score in adults [29,30] and the Pediatric Risk of Mortality score in children [31]. Need for mechanical ventilation, oliguria, and circulatory instability are the signs typically observed when IAH progresses to ACS. Since prerequisites for an ACS in animals have not been defined yet [32], a relevant animal model of ACS should display this 'classic triad' of an ACS [33-36]. In the present investigation, the ventilated animals displayed a decreased circulatory and renal function. Depending on the level of IAH, intestinal ischemia and BT to the intestinal wall, as well as to MLN, also occurred.

Depending on the level of IAH, the circulation in study pigs was compromised as evidenced by the significant decrease in volumetric preload indices and signs of ongoing acidosis. Accordingly, base excess was found to correlate very strongly with GEDVI. As proven in former studies using different techniques (e.g., flowmeters, near infrared spectroscopy, laser Doppler flow measurement [37-42]), IAH indeed suppresses arterial perfusion as well as venous outflow of intra- and extra-abdominal organs, resulting in tissue ischemia [43]. In the present study, the limited circulation was mirrored by marked decreasing APP and RFG values. RFG was found to correlate very strongly with urine output. The amount of histomorphological damage of the intestines increased dramatically with different IAH levels. Other investigations found comparable hemodynamic changes [44] and ischemic damage in the bowel specimen of pigs exposed to IAH over at least 18 h [8,9,14,45].

These disturbances even become aggravated by generalized edema and increasing interstitial pressures in thoracic as well as in abdominal tissues due to changed lymph flows. Systemic inflammation causes capillary barrier damages with increasing lymphatic flows which lead to an elevation of lymphatic pressures. As soon as the drainage capacity of the lymphatics is reached, the amount of edema in the pulmonary and splanchnic interstitium increases dramatically [46]. On the other hand, mechanical ventilation as well as IAH additionally impedes the lymphatic backflows from thoracic and abdominal tissues and compartments. The critical IAP point at which lymph flow starts to decrease and tissue water contents progressively increase seems to be likely around 15 mmHg [47]. With reference to our study, this might explain, why the so-called hemodynamic 'filling volumes' such as GEDVI decreased in pigs which belonged to the IAP-30 group, while in pigs belonging to the IAP-15 group, no significant changes were obtained. This might be explained by venous autotransfusion of visceral blood which could have counterbalanced lymphatic fluid losses in this experimental group. This autotransfusion is reflected by a significant increase of MAP in animals belonging to the IAP-15-group (see Table 4). Representing a parameter for impeded lymphatic outflow and increasing pulmonary edema, the EVLWI nearly tripled in IAP-30 pigs and approximately doubled in IAP-15 animals over the investigational period. Probably caused by mechanical ventilation and non-physiologically supine positioning, even controls showed an increase in EVLWI by 40%. In the same pig model, our study group found comparable changes of filling volumes when investigating the influence of an IAP of 30 mmHg over 24 h using the double-indicator dilution technique [25]. The higher the IAP increases, the more extravasation of fluids on one hand (thereby with arising intravascular dehydration) and a thickening of interstitial tissues on the other hand (thereby with arising elongation of diffusion courses) seem to additionally aggravate pressure-induced tissue ischemia and histomorphological damages.

Although recommended as a nonsurgical therapy option, changes of hemodynamics under the influence of IAH were observed but consciously not corrected by demand-adapted supply of additional fluids or vasoactive agents in order to investigate the consequences of intravascular volume depletion on BT. Further investigations are needed to elucidate whether comparable ischemic damage and BT would have been preventable in this model, if medical management algorithms were implemented and hemodynamics were maintained.

As a consequence of tissue ischemia, the 'mechanical barrier' as well as 'immunologic' and 'ecological' barrier functions of the bowel wall might break down and lead to 'bacterial overgrowth' with BT [11,48,49]. In the present study, increasing IAP resulted in more translocated bacteria in bowel wall specimens as well as in MLN. Thus, on a BT dissemination scale of 1 to 3 (presented by Sukhotnik [19]), we detected an IAH-induced level of up to 1 BT in both experimental groups ('local' BT). The presence of translocated bacteria in usually sterile organs or fluids (such as blood and lymph) is considered to amplify an inflammatory response of immunocompetent cells which already might have been activated by tissue ischemia. In the past, several animal models concerning gut-derived sepsis (regardless of IAP) proved the detrimental effects of BT on the amplification of systemic inflammatory response syndrome (SIRS) and the progression to multi-organ dysfunction (MODS) and MOF [10,50-52]. Our results support the assumption that IAH could also provoke MODS and MOF not only on the basis of mesenteric ischemia, but also especially by BT. Therefore, BT might serve as an accelerator of the resulting MODS [50].

Only three large-animal models have been performed in the past which investigated the effects of IAH on BT. Two of these investigations used porcine models [14,38]. In a dog model, Tug et al. were not able to prove BT when exposing dogs to an IAP of 15 mmHg for up to 2 h. However, depending on the IAH level, they found an increasing presence of sinus histiocytosis in MLN and interpreted this fact as an indirect sign of bacterial drainage [53]. Doty et al. exposed pigs to hemorrhage followed by an IAH of 30 mmHg. After 1 h of increased IAP, animals were decompressed and further monitored in order to observe the subsequent reperfusion phase. No additional BT was found in the test group [38]. Insufficient culture techniques were assumed to be responsible for the lack of detectable bacteria. The development of ACS, however, may take as long as 24 h after the onset of the underlying cause in humans [2]. The inability to detect BT in the investigations of Doty et al. and Tug et al., therefore, could have been caused by the fact that the investigational time of both examinations was too short to initiate sufficient IAH-induced tissue damage with subsequent BT.

Accordingly, Kubiak et al. [14] observed significant bacteremia in all pigs examined using a 'pathological model of ACS' [32] within an observational period of 48 h. In this experiment, blood cultures were positive for *Pseudomonas aeruginosa, Escherichia coli, Klebsiella pneumoniae*, and *Proteus mirabilis*. This was comparable to the bacteria found in our study. Such enterobacteriaceae usually colonize the gastrointestinal tract and therefore might translocate as shown by other study groups [54,55].

Several study groups found 'regional' (level 2 BT; [15]) and 'systemic' BTs (level 3 BT; [16,20]) in small-animal models (Tables 9 and 10). Small-animal models, however, appear to be less relevant as renal, cardiovascular, and gastrointestinal physiology and anatomy are not comparable to human adults [10]. Since intra-abdominal dimensions and cardiovascular physiology of rats and rabbits are more likely comparable to those of neonates and small infants [56], the above-mentioned results of small-animal models rather reflect the detrimental effects of IAH on children. Thus, it might be presumed that smaller individuals react more sensitively to IAH and might suffer earlier from systemic health implications.

**Table 9:** Literature overviews of currently published animal experiments concerning bacterial translocation under the influence of IAH

Author [Lit.] (year)	Model, weight	IAH performed via	IAH level	IAH length	Additional intervention	Detection of bacterial translocation (BT)	Microbiologically proven bacteria
Eleftheriadis et al. [16] (1996)	Rats, 210 to 290 g	CO_2_-pneumo-peritoneum	15 mmHg	1 h	Decomp.+ reperf. for 3 h or 18 h	3 h^b^: MLN, liver, spleen 18 h^b^: liver, spleen	*E. coli *and 'other bacteria'
Diebel et al. [17] (1997)	Rats, 300 to 350 g	CO_2_-pneumo-peritoneum	20 to 25 mmHg	1 h	Maintenance of MAP by additional fluids	Spleen^b^ n.s.: liver, spleen, peritoneum	*E. coli*, Enterobacter, Entercoccus, Pseudomonas and Staphylococcus spp.
Tug et al. [53] (1998)	Dogs^a^, 20 to 30 kg	CO_2_-pneumo-peritoneum	15 mmHg	0.5 or 2 h	-	ns.: PVB, MLN, liver, spleen, and peritoneum	Not given
Doty et al. [38] (2002)	Pigs^a^, 20 to 30 kg	Instillation of saline (intra-abdominal)	30 mmHg	1 h	1. Hemorrhage 30 min 2. IAH + high-vol. fluids 3. Decomp. + reperf. for 1 h	n.s.: PVB, MLN, spleen	PVB: *Staphylococcus aureus, E. coli*, Clostridium MLN: *E. coli*, Clostridium, *S. aureus, Escherichia fergusonii* Spleen: *S. aureus*
Polat et al. [18] (2003)	Rats, 200 to 250 g	CO_2_-pneumo-peritoneum	14, 20, or 25 mmHg	1 h	Decomp. + reperf. for 4 h	14 mmHg: n.s. 20 mmHg^b^: liver > MLN > spleen 25 mmHg^b^: spleen > > liver > > MLN	Gram (-) > Gram (+) Predominating bacterium: *E. coli*
Cheng et al. [15] (2003) (Chinese)	Rabbits	N_2_-pneumo-peritoneum	10, 20, or 30 mmHg	1, 2, or 4 h	-	10 mmHg: n.s. (1 h, 2 h, 4 h) 20 mmHg^b^: 33% (1 h), 67% (2 h), 100% (4 h) 30 mmHg^b^: 100% (1 h, 2 h, 4 h)	Not given
Yagci et al. [20] (2005)	Rabbits, 2.5 to 3.0 kg	Inflation of an intra-abdominal bag	10, 15, 20, or 25 mmHg	12 h	-	10 mmHg^b^: spleen 15 mmHg^b^: MLN 20 mmHg^b^: spleen > MLN > liver 25 mmHg^b^: MLN > spleen > liver	Gram (-) > Gram (+) Predominating bacteria: *K. pneumoniae, E. coli*, and *Serratia marcescens*
Sukhotnik et al. [19] (2006)	Rats, 250 to 300 g	Air-pneumo-peritoneum	15 or 25 mmHg	1 h	Decomp. + reperf. for 24 h	controls: 30% BT 15 mmHg^b^: 60% BT 25 mmHg^b^: 80% BT	*E. coli, S. aureus*, Enterococcus, Pseudomonas, Klebsiella spp., and *Morganella morganii*
Gong et al. [62] (2009)	Rats, 250 ± 50 g	N_2_-pneumo-peritoneum	20 mmHg	1 h	Decomp. + reperf. for 4 h	ACS^b^: MLN > liver > spleen ACS/De^b^: MLN > > liver > > spleen	Predominating bacterium: *E. coli*
Kubiak et al. [14] (2011)	Pigs^a^, 22 to 30 kg	Placement of fecal clot i.a. + clamping of superior mesenteric artery	20 mmHg and more	48 h	-	100% bacteremia (BT)	*P. aeruginosa, E. coli*, *K. pneumoniae, P. mirabilis*

^a^Large-animal models (in contrast to small-animal models). ^b^Significant difference compared to controls; n.s., no significant difference compared to controls. ACS, abdominal compartment syndrome (group); BT, bacterial translocation; CO_2_, carbon dioxide; De/decomp. + reperf., decompression and following reperfusion after IAH; Gram (+)/(-), Gram-positive or Gram-negative bacteria; IAH, intra-abdominal hypertension; MLN, mesenteric lymph nodes; PB, peripheral blood; PVB, portal vein blood; spp.: not further differentiated species of bacteria; vol.: administration of fluids (volume overload).

**Table 10:** Staging of bacterial transformation

Level	Distribution	Microbiological findings
Level 1	Local BT	Detection of gut-derived bacteria in mesenteric lymph nodes
Level 2	Regional BT	Detection of gut-derived bacteria in portal vein blood or liver
Level 3	Systemic BT	Detection of gut-derived bacteria in peripheral blood or other organs

Modified from Sukhotnik et al. [19]

### Study limitations

Although porcine models have been characterized as the best possible imitation for the physiology and anatomy of human adults, each animal model is known to have serious limitations. Reminded about the fact that BT and positive lymph nodes, as well as blood cultures, were also shown in animals belonging to the control group, the validity of the underlying porcine model must be judged carefully in studies concerning microbiological investigations. Not only typical gut-derived bacteria, but also Gram-positive bacteria which more likely might have been translocated sporadically from the lungs (e.g., Staphylococci, Streptococci) were found in all three groups. This is in accordance with the assessment that more than 90% of obviously healthy pigs histopathologically show signs of preexisting pulmonary infections [22,23]. Pavlikov stated that about two thirds of all bacteria which are cultivable in systemic lymph nodes of pigs might originate from pulmonary translocations [57].

Another essential limitation lies in the fact that the current study did not use a 'pathological model' as done by Kubiak et al. [14]. Pigs in their study developed a full-value ACS under the influence of intra-abdominal placement of fecal clots and by clamping the superior mesenteric artery which better reflects the pathophysiological circumstances found in patients suffering from IAH-inducing diseases or circumstances such as SIRS/sepsis with capillary leakage and the need for volume resuscitation which often leads to fluid overload. Contrary to that, the animals observed in our study only were forced with 'pure' pressures which directly or indirectly affected organs and their functions. In contrast to our model, Kubiak's approach appears 'multi-dimensional'. Nevertheless, we were able to prove that even without this additional systemic activation and provocation which come along with IAH-inducing diseases, within 24 h of IAH alone, the resulting ischemia and tissue damage is stimulating enough to break down mucosal barriers. As soon as additional stimuli supervene (such as underlying IAH-inducing diseases), these devastating processes potentially become aggravated and even accelerated.

Although the World Society of the Abdominal Compartment Syndrome recommended bladder filling volumes of no more than 25 mL when measuring IVP in subjects weighing at least 25 kg, we decided to use volumes of 50 mL in this model in order to be able to compare the results of the underlying study with those of earlier investigations [8,9]. Based on experimental results, Gudmunndsson et al. even stated to limit the amount of fluid in the bladder to 15 mL in his pig model in order to avoid an overestimation of IAP values [58]. Since pigs of our study weighed nearly twice as much when compared to those of Gudmundsson's study, these recommendations cannot be transferred one by one. Furthermore, the applicability of our IAP measurements was proven by separate testings [59].

Significant changes in arterial PO_2 _and PCO_2 _were not observed although CO_2 _was used to increase the IAP. Clinical and experimental studies have observed a peritoneal resorption of CO_2 _at an IAP of 15 mmHg [60], which theoretically could lead to an ongoing or aggravating acidosis. Regarding the underlying model, increases in PCO_2 _were only minor and counterbalanced by a slight hyperventilation, as the minute ventilation was set to yield a PCO_2 _of 35 to 40 mmHg under baseline condition. As soon as IAH exceeds 16 mmHg, the resorption of intra-abdominal CO_2 _is reduced due to the IAH-induced compression of peritoneal blood and lymph vessels [61]. This would explain why PCO_2 _remained essentially unchanged also in the IAP-30 group.

## Conclusions

In both treatment groups, IAH led to tissue ischemia and to a level 1 BT to deeper bowel wall layers and MLN. Even low IAH levels, e.g., 15 mmHg, result in remarkable amounts of translocated bacteria after 24 h. These findings are in line with observations that IAH might provoke sepsis with MODS. Thus, cases of persisting or progressing IAH require a clear therapy strategy to prevent the deleterious effects of subsequent ischemic damage, BT, and MOF.

## Abbreviations

ACS: abdominal compartment syndrome; ANOVA: analysis of variance for repeated measurements; APP: abdominal perfusion pressure; BE: base excess; BT: bacterial translocation; CFU: colony forming units; CO_2_: carbon dioxide; CI: cardiac index; CVP: central venous pressure; EVLWI: extra-vascular lung water index; GEDVI: global end-diastolic volume index; HPF: high-power field; HR: heart rate; IAH: intra-abdominal hypertension; IAP: intra-abdominal pressure; IVP: intravesical pressure; n.s.: not significantly different; MAP: mean arterial pressure; MLN: mesenteric lymph node; MODS: multiple organ dysfunction syndrome; MOF: multi-organ failure; PiCCO: pulse contour cardiac output; PVB: portal venous blood; RFG: renal filtration gradient; SD: standard deviation; UO: urine output.

## Competing interests

In addition to his assistant professorship at the RWTH Aachen University (Germany), Alexander Schachtrupp is head of the Department of Medical Sciences at B. Braun Melsungen in Germany. B. Braun does not distribute any medical devices or products concerning the diagnosis and/or treatment of IAH or ACS. The other authors declare that they have no competing interests.

## Authors' contributions

Literature research was done by TK and AS. Data collection was performed by all stated authors. The article was written by TK and AS. The article was revised by MA, PK, RTC, H and JC. Linguistic advice was provided by PK. All authors read and approved the final manuscript.
